# Systems biology of bacteriophage proteins and new dimensions of the virus world discovered through metagenomics

**DOI:** 10.1186/gb-2011-12-s1-p9

**Published:** 2011-09-19

**Authors:** David M Kristensen, Arcady R Mushegian, Eugene V Koonin

**Affiliations:** 1 National Center for Biotechnology Information, National Library of Medicine, National Institutes of Health, Bethesda, MD 20894, USA; 2 Department of Bioinformatics, Stowers Institute for Medical Research, Kansas City, MO 64110, USA; 3 Department of Microbiology, Molecular Genetics, and Immunology, University of Kansas Medical Center, Kansas City, KS 66160, USA

## 

Most of the DNA viruses in the gastrointestinal tract are phages, which infect bacterial hosts. Despite phages being the most abundant organisms on Earth, as well as extremely active players in the global ecosystem, much remains unknown about how they function in their natural environments. Advances in whole genome sequencing technologies have generated a large collection of hundreds of phage genomes, allowing deep insight into the genetic evolution of phages, and metagenomics technologies seem to promise more rewarding glimpses into their life cycles and community structures.

Recently, we developed an automated approach to assemble a collection of orthologous gene clusters of double-stranded DNA phages (phage orthologous groups, or POGs). This approach follows the well-known clusters of orthologous groups (COGs) framework to identify sets of orthologs by examining top-ranked sequence similarities between proteins in complete genomes without the use of arbitrary similarity cutoffs, and it thus represents a natural system for examining fast-evolving and slow-evolving proteins alike. This automated approach was designed to keep pace with the rapid and accelerating growth of whole genome information from sequencing projects. In particular, we employ a faster graph-theoretical COG-building algorithm that vastly improves our ability to deal with larger numbers of genomes (*N*) by reducing the worst-case complexity from *O*(*N*^6^) to *O*(*N*^3^ × log *N*). This system encompasses more than 2,000 groups from the almost 600 known phage genomes deposited at the National Center for Biotechnology Information and is in the process of being expanded to include single-stranded DNA phages and single- and double-stranded RNA phages.

Using this approach, we found that more than half of the POGs have no or very few evolutionary connections to their cellular hosts, indicating that these phages combine the ability to share and transduce the host genes with the ability to maintain a large fraction of unique, phage-specific, genes. Such genes are useful for targeted research strategies: for example, as diagnostic indicators and fundamental units of systems biology studies. We employed this set of phage-specific genes to probe the composition of several oceanic metagenomic samples. Although virus-enriched samples indeed contain more homologous matches to phage-specific POGs than a full metagenomic sample also containing cellular DNA, the total gene repertoire of the marine DNA virome is dramatically different from that of known phages. In particular, it is dominated by rare genes, many of which might be contained within viruslike entities such as cellular gene transfer agents rather than true viruses. This result might suggest the necessity of radically rethinking what constitutes the ‘virus world’, because the major component of (marine) viromes could be gene transfer agents that encapsidate bacterial and archaeal genes.

